# Up-Regulation of GITRL on Dendritic Cells by WGP Improves Anti-Tumor Immunity in Murine Lewis Lung Carcinoma

**DOI:** 10.1371/journal.pone.0046936

**Published:** 2012-10-15

**Authors:** Jie Tian, Jie Ma, Ke Ma, Bin Ma, Xinyi Tang, Samuel Essien Baidoo, Jia Tong, Jun Yan, Liwei Lu, Huaxi Xu, Shengjun Wang

**Affiliations:** 1 Department of Laboratory Medicine, The Affiliated People's Hospital, Jiangsu University School of Medical Science and Laboratory Medicine, Zhenjiang, China; 2 Tumor Immunobiology Program, James Graham Brown Cancer Center, University of Louisville, Kentucky, United States of America; 3 Department of Pathology and Centre of Infection and Immunology, The University of Hong Kong, Hong Kong, China; Ludwig-Maximilians-University Munich, Germany

## Abstract

**Background:**

β-Glucans have been shown to function as a potent immunomodulator to stimulate innate and adaptive immune responses, which contributes to their anti-tumor property. However, their mechanisms of action are still elusive. Glucocorticoid-induced TNF receptor ligand (GITRL), a member of the TNF superfamily, binds to its receptor, GITR, on both effector and regulatory T cells, generates a positive co-stimulatory signal implicated in a wide range of T cell functions, which is important for the development of immune responses.

**Methodology/Principal Findings:**

In this study, we found that whole β-glucan particles (WGPs) could activate dendritic cells (DCs) via dectin-1 receptor, and increase the expression of GITRL on DCs *in vitro* and *in vivo*. Furthermore, we demonstrated that the increased GITRL on DCs could impair the regulartory T cell (Treg)-mediated suppression and enhance effector T cell proliferation in a GITR/GITRL dependent way. In tumor models, DCs with high levels of GITRL were of great potential to prime cytotoxic T lymphocyte (CTL) responses and down-regulate the suppressive activity of Treg cells, thereby leading to the delayed tumor progression.

**Conclusions/Significance:**

These findings suggest that particulate β-glucans can be used as an immunomodulator to stimulate potent T cell-mediated adaptive immunity while down-regulate suppressive immune activity via GITR/GITRL interaction, leading to a more efficient defense mechanism against tumor development.

## Introduction

β-Glucans are well-known biological response modifiers (BRMs) that have been used to treat cancer for many years with varying and unpredictable efficacy. β-Glucans are extracted from the cell wall of fungi, yeast or bacteria, consisting of β-1,3-linked D-glucopyranosyl residues with β-1,6-linked D-glucopyranosyl side chains of varying length and distribution frequency [1]. Over the past decades, β-glucans have been recognized as pathogen-associated molecular patterns (PAMPs) by innate immune system. Thus far, there are four β-glucan receptors that have been identified: complement receptor 3 (CR3, CD11b/CD18, Mac-1, αMβ2 integrin) [2], dectin-1 [3], [4], [5], lactosylceramide (LacCer) [6] and scavenger receptor (SR) [7]. However, dectin-1 is the main receptor mediating the activity on macrophages and dendritic cells (DCs) [8], [9]. Engagement of dectin-1 by β-glucan can trigger a series of intracellular signal transduction pathways through Syk kinase and Raf-1signaling pathway, activating the cells and inducing a variety of cellular responses, such as cytokine production [10], [11], [12]. Recent findings have demonstrated that β-glucans are capable of functioning as a potent adjuvant to stimulate innate and adaptive immune responses [3], [13], [14], [15].

Glucocorticoid-induced TNFR family-related receptor (GITR) and its ligand, GITRL, are members of the TNFR/ligand superfamilies [16]. GITR is at low levels on conventional T cells and is up-regulated upon activation of CD4^+^ T cells and CD8^+^ T cells, but it is particularly expressed at high levels on CD4^+^CD25^+^ regulatory T cells (Tregs). The high level of GITR expression by Treg cells indicates its essential role for this subset [17], [18], [19], [20], [21]. GITRL is expressed on the surface of various antigen-presenting cells (APCs), including DCs, macrophages and B cells [22], [23]. GITR/GITRL interplay is considered in a wide range of immune functions involving both effector and Treg cells [21], [24]. Indeed, GITR engagement on effector T cells by agonistic Abs or recombinant GITRL *in vitro* exhibits positive co-stimulatory signals for TCR-stimulated T cell activation, leading to increased T cell proliferation and cytokine production [17], [18], [22], [25], [26]. Additionally, triggering of GITR on Treg cells in co-culture with effector T cells has been suggested to abrogate the suppressive capacity of Treg cells [19], [27]. However, some other studies indicate that GITR ligation on Treg cells does not affect the suppressive activity of Tregs themselves, but the engagement of GITR on effector T cells allows them to escape suppression by regulatory T cells [22]. In conclusion, the GITR/GITRL interaction proves to be an effective approach to manipulate the activity of both effector T cells and Treg cells, which is suggested to be an essential therapeutic target.

In this study, we demonstrated that whole β-glucan particles (WGPs) could activate and maturate DCs, and up-regulate the GITRL expression on DCs both *in vitro* and *in vivo*, thus promoting the proliferation of CD4^+^CD25^−^ effector T cells, leading to augmented cytotoxic T lymphocyte (CTL) responses via GITR/GITRL interaction in tumor models. Furthermore, we showed that the engagement of GITR/GITRL could also impair Treg cell-mediated suppression *in vitro* and abrogate peripheral Treg suppressive capacity in tumor-bearing mice. More importantly, the tumor infiltrated Treg cells were reduced, suggesting a localized abrogation of suppression. All these effects promote anti-tumor immunity and provide a more efficient defense mechanism against tumor development.

## Results

### WGP induces the up-regulation of GITRL on BMDCs via dectin-1

**Figure 1:**
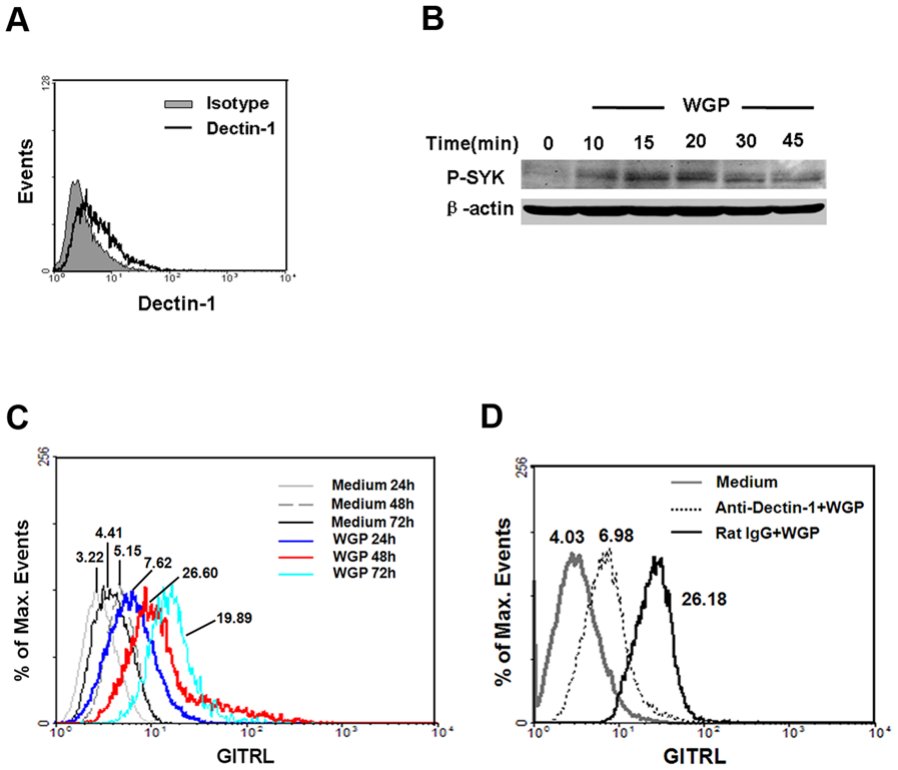
Up-regulation of GITRL on BMDCs via dectin-1 upon WGP stimulation. (A) Expression of dectin-1 on BMDCs. BMDCs were stained for dectin-1 with anti-dectin-1 antibody (thick line) or rat IgG2b (solid gray) and then analyzed using flow cytometry. (B) Analysis of the activation of SYK by immunoblot of lysates of BMDCs stimulated with WGP (time, above lanes), assessed with anti-phospho-SYK antibody (upper band). β-actin levels were measured as protein loading controls (lower band). (C) Flow cytometry for surface expression of GITRL on BMDCs after no stimulation or stimulation with WGP for 24 h, 48 h and 72 h. (D) BMDCs were pretreated with anti-dectin-1 mAb or rat IgG (5 µg/ml) for 1 h at 37°C and then treated with 100 µg/ml WGP, after 48 h stimulation, cells were subjected to analyze the GITRL expression. The values shown in the flow cytometry profiles are geometric mean fluorescent intensity. Data are representative of three independent experiments.

First, we investigated the expression of dectin-1 on BMDCs. Flow cytometry analysis showed that BMDCs expressed dectin-1 (Figure 1A). The geometric mean fluorescence intensity (Geo MFI) of dectin-1 on BMDCs was 6.01±0.99, while isotype was 3.31±0.18. In order to investigate whether the downstream signaling molecule SYK could be activated in BMDCs after WGP stimulation, SYK was assayed at different time points upon WGP treatment. As indicated in Figure 1B, WGP stimulation induced SYK activation, and a significant up-regulation of SYK phosphorylation in BMDCs was at about 15–20 min post stimulation (P-SYK/β-actin IOD: 0.0078±0.0018 *vs* 0.0654±0.0075, P<0.001). Next, we determined the expression of GITRL on BMDCs after WGP stimulation and found that the GITRL level was dramatically increased at 48 h (red line, Geo MFI: 26.60) upon WGP treatment (Figure 1C). To further investigate whether the increase of GITRL was mediated by dectin-1, anti-dectin-1 antibody was used for blocking. Addition of anti-dectin-1 antibody in the presence of WGP-stimulated BMDCs partially reversed the effect that WGP induced while the control IgG did not. As indicated in Fig. 1D, after the dectin-1 was inhibited, GITRL expression was down-regulated. In addition, we studied the expression of other co-stimulatory molecules, including CD40, CD80, CD86 and MHCII in WGP-stimulated BMDCs and found that the expression of CD40, CD80, CD86 and MHCII was significantly increased (data not shown). Taken together, WGP could induce the activation and maturation of DC through dectin-1, and up-regulate GITRL expression on them *in vitro*.

### WGP-induced enhancement of GITRL impairs Treg cell-mediated suppression and enhances the effector T cell proliferation

**Figure 2:**
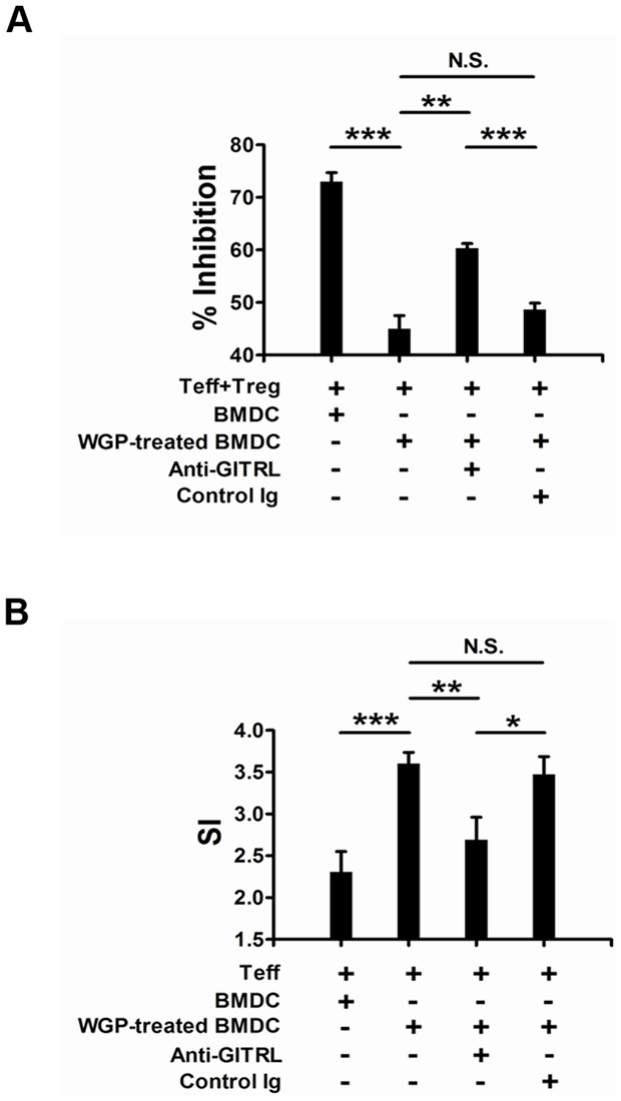
Increased GITRL expression on BMDCs by WGP impairs Treg-mediated suppressive effect and enhances Teff proliferation. Murine CD4^+^CD25^−^ Teff and CD4^+^CD25^+^ Treg cells were purified from C57BL/6 mice splenocytes as described in *Materials and Methods*. BMDCs were stimulated with WGP (100 µg/ml) or not for 24 h. Cells were harvested, treated with mitomycin C and then co-cultured at a 1∶5 ratio with CD4^+^CD25^−^ Teff and CD4^+^CD25^+^ Treg cells (A) or Teff cells (B) in the presence of anti-CD3 mAb with or without anti-GITRL blocking antibody or control Igs for 72 h, Treg and Teff cells were added at 1∶1 ratio. Wells were pulsed with 1 µCi/well [^3^H]-thymidine for the last 16 h. Data are expressed as means ± SD. ***P<0.001, **P<0.01, *P<0.05. N.S. represents no significance between columns.

It has been suggested that GITR/GITRL interaction functions as a co-stimulating signal and modulates T cell activation and function [28]. Having observed the up-regulated expression of GITRL on BMDCs by WGP, we next investigated whether the increased GITRL would alter the activation or function of T cells via GITR/GITRL interplay. First, we co-cultured CD4+CD25+ Treg cells and CD4+CD25− Teff cells with WGP-treated BMDCs or non-treated BMDCs. As shown in Figure 2A, significant reduction of percentage of inhibition was observed in the presence of WGP-treated BMDCs when compared with non-treated cells. To further investigate whether the reduction of inhibition was caused by the up-regulation of GITRL on BMDCs and GITR/GITRL interaction in part mediated this response, anti-GITRL antibody was used for blocking. Addition of anti-GITRL antibody in the presence of WGP-stimulated BMDCs partially reversed the inhibition extent while the control Ig did not. This suggests that the Treg cell-mediated suppression might be inhibited by GITR/GITRL interplay. Next, we co-cultured Teff cells with WGP-stimulated BMDCs or non-treated cells and found that the proliferation of Teff cells was efficiently enhanced in the presence of WGP-treated BMDCs. However, the enhanced proliferation response was inhibited by the addition of anti-GITRL antibody (Figure 2B). Therefore, the enhancement of GITRL on BMDCs can regulate the suppressive effect induced by Treg cells and enhance the proliferation of Teff cells which are GITR/GITRL pathway dependent.

### WGP treatment *in vivo* significantly enhances GITRL expression on DCs and delays tumor progression

**Figure 3:**
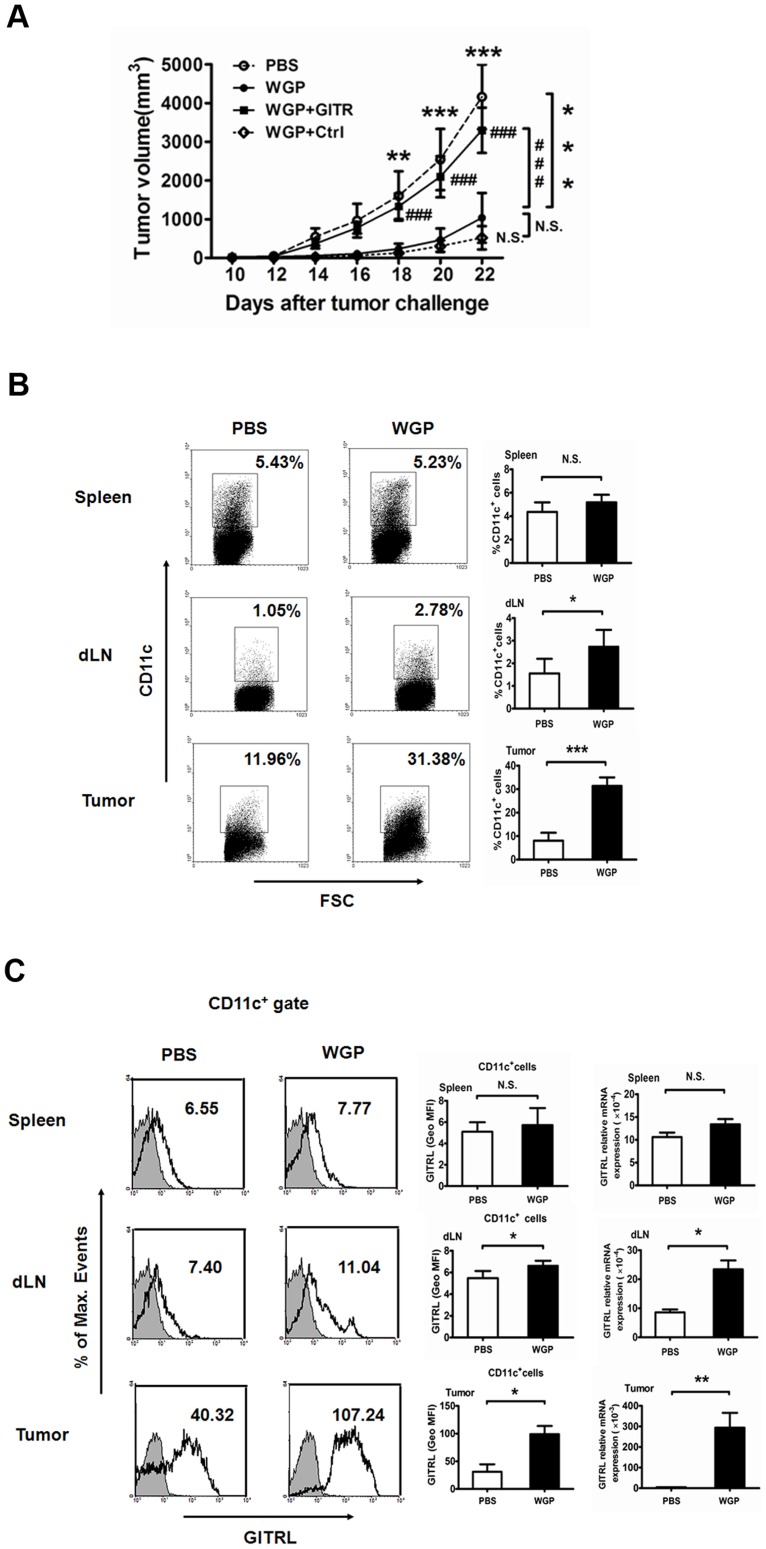
WGP treatment up-regulates GITRL expression on DCs in tumor-bearing C57BL/6 mice. (A) Groups of mice (n = 6) bearing established Lewis lung carcinoma were treated as described in *Materials and Methods*. Tumors were measured with a caliper at indicated time. (B) The proportions of CD11c^+^ DCs in spleens, draining lymph nodes (dLN) and tumor tissues from WGP-treated or untreated mice were analyzed using flow cytometry. (C) Expression of GITRL on CD11c^+^ cells in spleens, draining lymph nodes and tumor sites treated with or without WGP were assessed by flow cytometry. Histograms are gated on CD11c^+^ cells (isotype: solid gray). RNAs extracted from spleens, draining lymph nodes and tumors were subjected to qRT-PCR for GITRL expression. Numbers indicate percentages of events in gate (dot plot) or geometric mean fluorescence intensity (GeoMFI; histograms). Results are expressed as mean ± SD. ***P<0.001, **P<0.01, *P<0.05, ^###^P<0.001, N.S. represents no significance between columns.

Having observed that WGP could increase the GITRL expression on BMDC *in vitro*, we next investigated whether WGP treatment would regulate the GITRL *in vivo* and have any effect on tumor therapy. To this end, C57BL/6 mice orally administered with or without WGP for 7 days were implanted with LLC tumor cells. Mice were continuously treated with or without WGP for another 3 weeks. As shown in Figure 3A, tumor-bearing mice treated with WGP exhibited a significantly slower tumor progression as compared to those treated with PBS. To further evaluate whether the delayed tumor development was caused by the increased GITRL by WGP treatment, we used GITR to block the GITR/GITRL interaction *in vivo*. It was obvious that tumor grew faster after GITR treatment while the control protein did not. It suggests that WGP could delay the tumor progression by up-regulating GITRL *in vivo*.

We next examined the expression of GITRL on DCs in tumor-bearing mice. Cells from spleens, draining lymph nodes and tumors in tumor-bearing mice treated with or without WGP were subjected to analyze the GITRL expression on DCs by flow cytometry. As shown in Figure 3B and 3C, the proportions of DCs in draining lymph nodes and tumors, and GITRL expression on DCs were significantly increased upon WGP treatment. In addition, GITRL mRNA levels in draining lymph nodes and tumors were dramatically up-regulated in WGP-treated group. All these data indicate that WGP treatment is able to increase the GITRL expression on DCs *in vivo* and subsequently slow tumor development.

### Augmented CTL responses are induced by up-regulation of GITRL following WGP treatment

**Figure 4:**
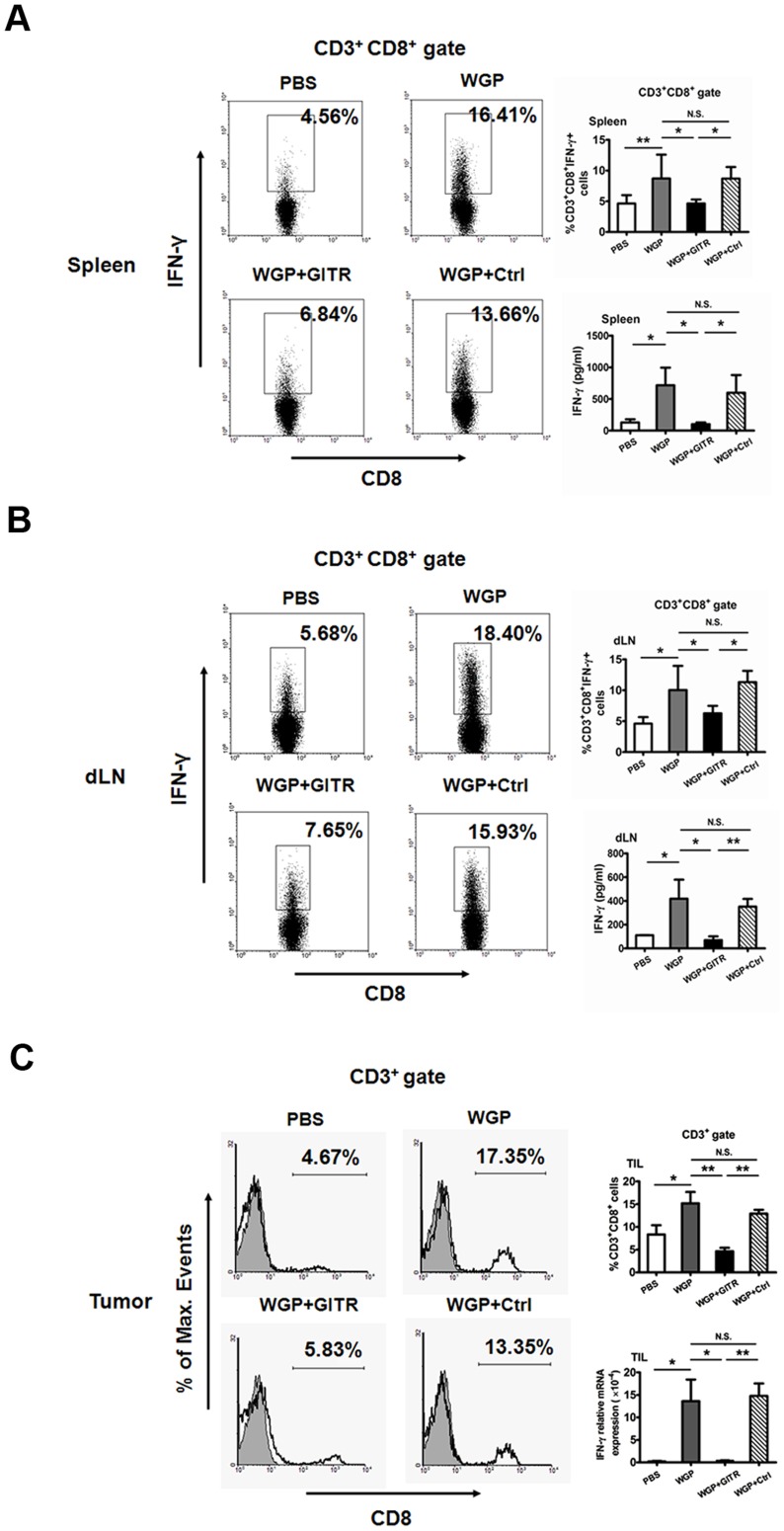
WGP induces enhanced CTL priming *in vivo*. Groups of mice (n = 6) bearing established Lewis lung carcinoma were treated as described in *Materials and Methods*. (A, B) Single cell suspensions prepared from spleens (A) and draining lymph nodes (B) were stimulated with PMA plus ionomycin and stained intracellular IFN-γ. Cells were gated on CD3^+^CD8^+^ T cells. Culture supernatants from splenocytes and lymphoid cells were collected and assayed for IFN-γ using ELISA. (C) Tumor specimens from each group were prepared for single cell suspensions. Cells were stained with mAbs against CD3, CD8 and were assessed by flow cytometry. Cells were gated on CD3^+^ T cells. RNAs from tumor specimens were extracted and qRT-PCR was performed for IFN-γ. Results are expressed as mean ± SD. **P<0.01, *P<0.05, N.S. represents no significance between columns.

We further determined the potential of enhanced GITRL on DCs to prime CD8^+^ T cells. Lymphocytes from spleens, draining lymph nodes and tumors in tumor-bearing mice treated with or without WGP were analyzed. Increased proportions of CD3^+^CD8^+^ T cells in spleens and draining lymph nodes were observed after WGP treatment (data not shown). As depicted in Figure 4, augmented CD8^+^IFN-γ^+^ CTLs in spleens (Figure 4A) and draining lymph nodes (Figure 4B) were induced in response to WGP treatment. Moreover, production of IFN-γ in culture supernatants from splenocytes and lymphoid cells was increased in WGP-treated group as compared to PBS control. Further, in local tumor sites, proportions of CD3^+^CD8^+^ T cells and IFN-γ mRNA levels were enhanced in WGP-treated mice (Figure 4C). However, the augmented CTL responses were reversed after the blocking treatment with GITR, which further suggests that WGP could boost the CTL responses in a GITR/GITRL dependent way.

### Increased GITRL induced by WGP inhibits the suppressive function of regulatory T cells in tumor-bearing mice

**Figure 5:**
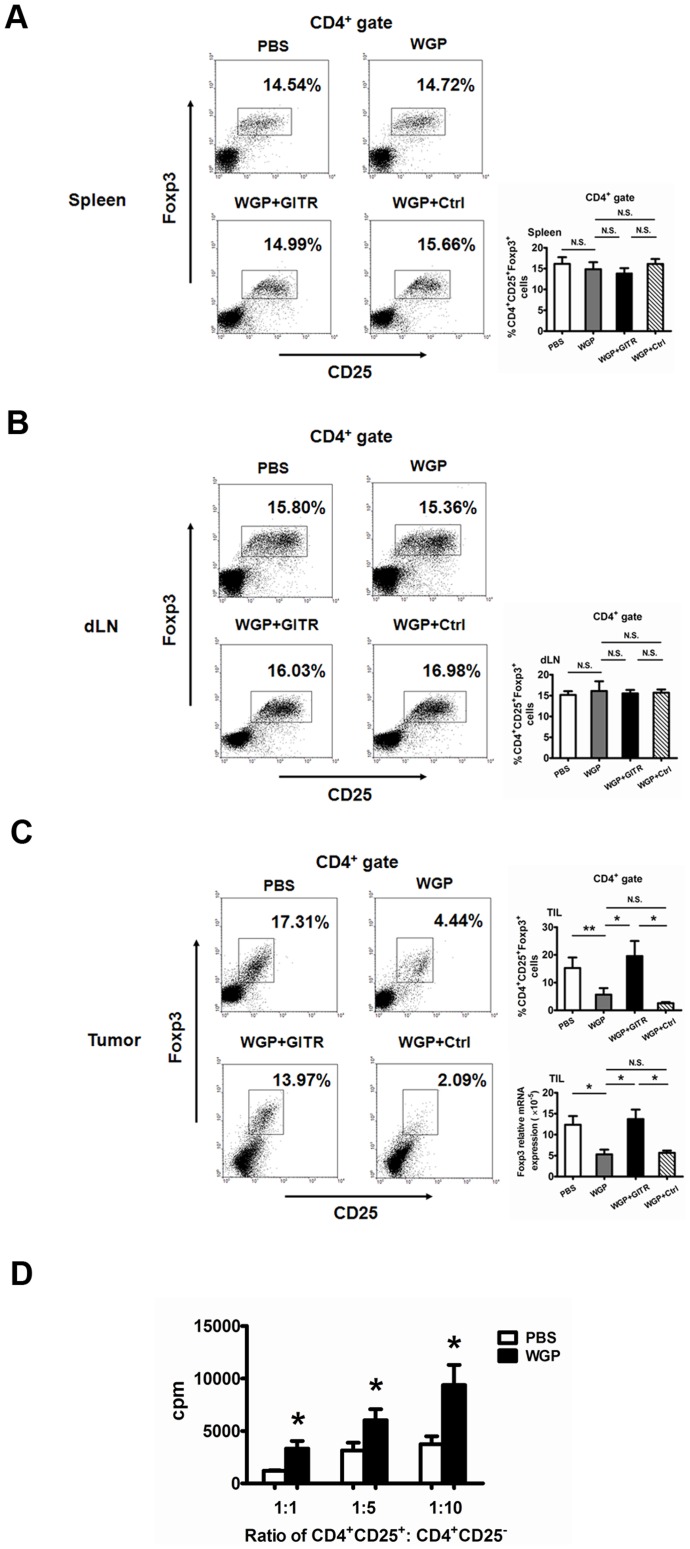
WGP alters the suppressive capacity of regulatory T cells in spleens. Groups of mice (n = 6) bearing established Lewis lung carcinoma were treated as described in *Materials and Methods*. (A, B, C) Single cell suspensions from spleens (A), draining lymph nodes (B) and tumor tissues (C) were stained with fluorochrome labeled mAbs to evaluate the proportions of CD4^+^CD25^+^Foxp3^+^ Tregs. Cells were gated on CD4^+^ T cells. RNAs from tumor specimens were extracted and Foxp3 mRNA expression was analyzed by qRT-PCR. (D) Splenic CD4^+^CD25^+^ Tregs isolated from tumor-bearing mice treated with or without WGP were co-cultured with CD4^+^CD25^−^ Teffs from wild type C57BL/6 mice in the presence of anti-CD3 mAb and anti-CD28 mAb for 72 h. Wells were pulsed with 1 µCi/well [^3^H]-thymidine and analyzed as described in *Materials and Methods*. **P<0.01, *P<0.05, N.S. represents no significance between columns.

Our *in vitro* experiment has indicated that the increased GITRL induced by WGP could inhibit the suppressive effect of Treg cells. Next, we determined whether the enhanced GITRL would have any effect on the activation and function of Treg cells in tumor models. As shown in Figure 5C, the percentages of CD4^+^CD25^+^Foxp3^+^ Treg cells infiltrated in tumor sites were dramatically decreased after WGP treatment, and were again reversed to high levels after GITR blocking treatment. In contrast, the proportions of Treg cells in spleens and draining lymph nodes were not altered after WGP treatment (Figure 5A and 5B). Strikingly, the suppressive activity of splenic Treg cells was substantially down-regulated after WGP treatment *in vivo* (Figure 5D). All these data suggest that, GITR/GITRL interaction could abrogate suppressive cells in local sites. In addition, although the engagement of GITR on splenic Treg cells failed to affect the proportions of peripheral Treg cells, it modulated the suppressive capacity of Treg cells.

## Discussion

β-Glucans have shown great success in cancer treatment as immunotherapeutic agents for centuries. β-Glucans are reported to have significant therapeutic efficacy in murine breast, liver metastasis, lung, and lymphoma tumor models as well as in human lymphoma and neuroblastoma when used in combination with anti-tumor mAbs [29], [30], [31], [32], [33]. Previous studies have demonstrated that β-glucans play an essential role in innate immune responses, and the mechanism in cancer therapy was mainly via stimulation of macrophages and priming of neutrophil complement receptor 3 for eliciting CR3-dependent cellular cytotoxicity of iC3b-opsonized tumor cells [34], [35], [36]. However, recent studies have suggested that β-glucans also have a critical role in regulating adaptive immune responses. It is reported that bacterial β-glucan curdlan-stimulated DCs could prime Th1, CTL and Th17 cells and convert Treg into IL-17-producing T cell via dectin-1 dependent pathway [37], [38]. In addition, recent studies reveal that yeast zymosan β-glucan appears to induce regulatory APCs leading to Treg differentiation [39], [40]. Moreover, a new report indicates that the particulate yeast-derived β-glucans WGPs can stimulate DC activation and maturation both *in vitro* and *in vivo*, leading to augmented Ag-specific CD4 and CD8 T cell responses, which bridges innate and adaptive immunity [15]. All these new emerging data suggest β-glucans could drive all arms of adaptive immune responses as potential immunomodulatory agents. In this study, we found that yeast-derived particulate β-glucans could activate DCs through dectin-1 to up-regulate the co-stimulatory molecule GITRL *in vitro*. Moreover, WGP *in vivo* treatment significantly increased the proportions of DC in tumor-bearing mice, especially in local tumor sites, and up-regulated GITRL expression on DCs, thus promoting the maturation of DCs, enhancing their capacity to activate adaptive T cell responses and eliciting anti-tumor immunity.

Since GITR was discovered in 1997, it has been the focus of many studies that investigate its biological function in cellular immunology [16], [17]. GITR is constitutively expressed on non-activated T cells and is especially abundant on CD4^+^CD25^+^ Treg cells. GITR activation, upon interaction with its ligand GITRL, functions as a co-activating signal [21]. Collectively, GITR triggering has two distinct effects. One is activating effector T cells, promoting their proliferation and making them more resistant to the Treg cell-mediated suppression. The other is abrogating the suppressive function of Treg cells or deletion of Treg cells. As our *in vitro* proliferation assays showed, the increased GITRL by WGP stimulation could impair the suppressive effect of Treg cell mediated and enhance the proliferation of effector T cells. This notion was further supported by adding agonistic anti-GITRL antibody to co-cultures of suppressor and responder T cells which led to a reversal of suppressive activity. Actually, upon the activation of DCs, apart from the GITRL enhancement, some other co-stimulatory molecules were also up-regulated, including CD40, CD80, CD86 and MHCII (data not shown). As shown in Figure 2, inhibition of GITR/GITRL interaction couldn't completely reverse the effect. Nevertheless, the role of GITR/GITRL played in the co-culture system was relatively dominant. In Lewis lung carcinoma model, orally administered WGP treatment significantly enhanced the GITRL on DCs *in vivo*. Therefore, those activated DCs with increased GITRL modulated anti-tumor responses from two sides, one was eliciting enhanced CTL responses, the other was down-regulation the suppressive capacity of Treg cells, which were both GITR/GITRL dependent. Mechanisms for immune suppression include both failure of immune T cell infiltrating into tumors and the presence of suppressive cells at tumor sites, such as regulatory T cells. Therefore, the most challenge task in tumor immunotherapy is to reverse the suppressive tumor microenvironment. In our study, we showed that particulate β-glucan WGPs modulated the tumor microenvironment towards anti-tumor responses via actively recruiting plenty of DCs with high GITRL expression into the tumor milieu, thus, eliciting augmented anti-tumor CD8 T cell responses and reducing the Treg cells infiltrated in tumors. Taken together, WGPs alter the suppressive environment and promote the effective adaptive immune responses via GITR/GITRL interaction, therefore, leading to an efficient approach to treat cancers.

One of the most efficient approaches in cancer immunotherapy is elicitation of potent anti-tumor T cell responses while down-regulation of immunosuppressive factors in patients with cancer. Actually, adjuvants have been widely used in cancer immunotherapy to potentiate anti-tumor T cell responses and modulate the activity of suppressive cells. Moreover, GITR/GITRL interaction has been proved to be an effective way to manipulate the T cell activity. In our study, we demonstrate that WGP could activate DCs via dectin-1 and up-regulate the GITRL expression on DCs, thus the GITR/GITRL interaction acts as a co-activating signal to augment efficient anti-tumor T cell responses and drastically alter the suppressive effect of regulatory T cells, leading to delayed tumor development. We thus provide novel insights into the mechanism of WGP in modulating adaptive immune responses and its function in anti-tumor immunity. Our findings highlight the benefit of particulate β-glucan treatment in cancer patients as an adjuvant and its potential for clinical application by promoting the effect of cancer immunotherapy.

## Materials and Methods

### Particulate yeast-derived β-glucan WGP

WGP (Eagan) was highly purified from the cell wall of *Saccharomyces cerevisiae* and was composed mainly of β-1,3/1,6-glucan. The particulate β-glucan WGP contains >85% β-glucan, <3.5% protein, <0.01% manan, and <8% moisture. To remove any traces of LPS contamination, the WGP was suspended in 200 mM NaOH for 20 minutes at room temperature (RT), then washed thoroughly, and finally re-suspended in LPS-free water [41]. The endotoxin level was 0.06 EU/mL as tested by the gel-clot method (Associates of Cape Cod).

### Bone marrow-derived DC (BMDC)

BM cells were isolated by flushing femurs and tibiae with complete RPMI 1640 medium using a 1 ml syringe. The complete RMPI 1640 medium was prepared by adding 10% heat-inactivated newborn calf serum (NCS) (GIBCO), 50 µM 2-mercaptoethanol and penicillin/streptomycin to RPMI 1640 medium (GIBCO). BM cells were seeded into 6-well plates (Corning) at 2.5×10^6^/ml in complete RPMI 1640 medium supplemented with 10 ng/ml granulocyte-macrophage colony-stimulating factor (GM-CSF) (Clongene Biotech) and 10 ng/ml IL-4 (PeproTech). Cells were cultured under 37°C, 5% CO_2_ in a humidified atmosphere. On day 2, medium was replaced with fresh medium supplemented with GM-CSF. On day 5, half of the medium was removed and fresh GM-CSF-supplemented medium was added into cultures. On day 7, the non- and loosely adherent cells were harvested and used for experiments.

### Western blot analysis

Proteins extracted from cells were prepared as described previously [42]. Proteins were separated by sodium-dodecyl-sulfate-polyacrylamide gel electrophoresis (SDS-PAGE), transferred onto immobilon PVDF membranes (Bio-Rad), and probed with rabbit phospho-SYK antibody (Cell Signaling Technology) and mouse β-actin antibody (Abcam) followed by chemiluminescent detection (Champion Chemical).

### *In vitro* proliferation assays

In our *in vitro* experiments, CD4^+^CD25^+^ Treg cells and CD4^+^CD25^−^ T cells were isolated from wild-type C57BL/6 mice splenocytes with a CD4^+^ T cell negative selection kit (Invitrogen), FITC-conjugated anti-CD25 mAb (BD Pharmingen) and anti-FITC microbeads (Miltenyi Biotec). The purity of isolated CD4^+^CD25^+^ Treg cells and CD4^+^CD25^−^ T cells was >90% (data not shown). BMDCs were pretreated with or without WGP for 24 h, and then the cells were harvested and treated with mitomycin C (Kyowa, 50 µg/ml) for 20 min. For co-stimulation assays, 5×10^4^ CD4^+^CD25^−^ T cells were co-cultured with 1×10^4^ mitomycin C-treated BMDCs in the presence of 10 µg/ml anti-CD3 mAb in triplicate in round-bottom 96 wells. For Treg assays, CD4^+^CD25^+^ Treg cells were added to the same conditions as described above at a Treg∶Teff ratio of 1∶1. Where indicated, 100 µg/ml anti-GITRL antibodies were added into the wells for blocking. In tumor models, CD4^+^CD25^+^ Treg cells from the spleens of WGP-treated or untreated tumor-bearing C57BL/6 mice were co-cultured with CD4^+^CD25^−^ T cells from wild-type C57BL/6 mice spleens, CD4^+^CD25^−^ T cells were plated at 5×10^4^ as responder cells with CD4^+^CD25^+^ suppressor cells at the following ratios of suppressor cells: responder cells 1∶1, 1∶5, 1∶10. Cells were incubated in the presence of anti-CD3 (10 µg/ml) and anti-CD28 (Biolegend, 5 µg/ml) mAbs. In all settings, cells were incubated for 72 h and pulsed with [^3^H]-thymidine (Pharmacia, 1 µCi/well) for the last 16 h of culture. Data from the co-culture were expressed as the % inhibition = [1−(cpm(CD4^+^CD25^−^ plus CD4^+^CD25^+^)/cpm CD4^+^CD25^−^)]×100 or stimulation index (SI) = cpm in stimulated culture/cpm in unstimulated culture [43]. % inhibition represented the inhibition of suppression and the uninhibited proliferation of Teff cells was used as 100%.

### Cell line, mice and tumor models

The Lewis lung carcinoma (LLC) cells were obtained from American Type Culture Collection. Specific pathogen free male C57BL/6 mice were purchased from Yangzhou University. All experiments were approved by the Institutional Committee on the Use of Animals for Research and Teaching,Jiangsu University.

For tumor models, C57BL/6 mice were treated with 200 µl of yeast-derived particulate β-glucan WGP (4 mg/ml in PBS; total 800 µg) or 200 µl PBS given every other day with an intragastric gavage needle for 7 days. Then, mice were implanted subcutaneously (s.c.) with LLC cells (3×10^6^/mouse). After tumor challenge, therapy was continuously administered for 3 weeks. Where indicated, GITR (500 µg/mouse) or control protein was administered intraperitoneally (i.p.) every other day to tumor-bearing mice on 2 day after the implantation of LLC cells. Tumor growth was monitored with bidirectional tumor measurements using calipers every 2 days and tumor volume was calculated using the formula V = 0.5ab^2^ with “a” as the larger diameter and “b” as the smaller diameter.

### Preparation of single cell suspension from tumors

Tumors were weighed and minced into small (1–2 mm^3^) pieces and immersed in 10 ml of digestion mixture including 5% NCS in RPMI 1640, 0.5 mg/ml collagenase A (Sigma-Aldrich), 0.2 mg/ml hyaluronidase (Sigma-Aldrich), and 0.02 mg/ml DNase I (Sigma-Aldrich) per 0.25 g of tumor tissues. This mixture was incubated at 37°C for 1.5 h on a rotating platform. Then the cell suspensions were filtered through 70 µm cell strainers (BD Falcon) and washed twice with ice cold PBS. Remaining red blood cells were lysed in ammonium chloride solution.

### Tumor infiltrated lymphocytes (TILs) isolation

TILs were isolated from tumor suspensions by density gradient centrifugation using Percoll (GE health). Briefly, cells were suspended in 80% Percoll, overlayed with 40% Percoll, and centrifuged at 2000×g for 30 minutes. Cells at the interface were collected [44].

### Flow cytometry

Spleens and draining lymph nodes were grinded with the 5 ml plunger of syringe, then the cell suspensions were filtered through 70 µm cell strainers (BD Falcon) and washed with ice cold PBS. Remaining red blood cells were lysed in ammonium chloride solution. Cells were harvested, Fc receptors were blocked by incubation in HB197 supernatant, stained with relevant antibodies in PBS for 30 min at 4°C. For surface markers, single cell suspensions were stained with FITC-conjugated CD11c and PE- conjugated GITRL mAbs (eBioscience). Anti-dectin-1 (Invivogen) and FITC-conjugated goat anti-rat IgG (KPL) were used to detect the dectin-1 expression. For intracellular cytokine staining, single cell suspensions were stimulated with PMA (Sigma-Aldrich, 50 ng/ml), ionomycin (Enzo, 1 µg/ml), monensin (Enzo, 2 µg/ml). After 5 h, cells were stained with anti-CD3 and anti-CD8 mAbs (eBioscience), fixed, permeabilized and stained with anti-IFN-γ mAb (eBioscience) according to the Intracellular Staining Kit (Invitrogen) instructions. For Treg cell staining, anti-CD4, anti-CD25 and anti-Foxp3 mAbs (eBioscience) were performed following Foxp3 Staining Buffer Set (eBioscience) protocols. Flow cytometry was performed using FACSCalibur Flow Cytometer (Becton Dickinson) and data were analyzed using WinMDI 2.8 software.

### Quantitative real-time PCR (qRT- PCR)

Total RNA was prepared with TRIzol (Invitrogen). To remove the possible contamination of genomic DNA, we treated the total RNA with DNase I (TaKaRa) before reverse transcription. cDNA was synthesized with ReverTra Ace qPCR RT kit (TOYOBO) according to the manufacturer's instructions. The indicated mRNA levels were quantified by qRT-PCR amplification using the Rotor-Gene 6000 (Corbett Research). Briefly, the cDNA was amplified in a 25 µl reaction mixture containing 12.5 µl SYBR Green Mix (TaKaRa), 200 nM of each primer, 100 ng of cDNA using the recommended cycling conditions. primer sequences were as follows: β-actin, 5′-TGGAATCCTGTGGCATCCATGAAAC-3′ (forward), 5′-TAAAACGCAGCTCAGTAACAGTCCG-3′ (reverse); GITRL, 5′- CTACGGCCAAGTGATTCCTGT -3′ (forward), 5′-GATGATCCCCCAGTATGTGTT -3′ (reverse); IFN-γ, 5′-CGCTACACACTGCATCTTGG -3′ (forward), 5′-TGAGCTCATTGAATGCTTGG -3′ (reverse); Foxp3, 5′-CCACTGGGGTCTTCTCCCTCAA -3′ (forward), 5′-CATTTGCCAGCAGTGGGTAGGA -3′(reverse). Relative quantification of mRNA expression was calculated by the comparative threshold cycle (Ct) method [45].

### Enzyme-linked immunosorbent assay (ELISA)

IFN-γ content in the supernatants from splenocyte and lymphoid cell cultures were measured by sandwich ELISA (eBioscience).

### Statistical analysis

The statistical significance of differences between groups was determined by the Student's *t* test or two-way analysis of variance. All analyses were performed using SPSS11.5 software. Differences were considered significant at a P level less than 0.05.
